# Fetal outcome in the critically ill pregnant woman

**DOI:** 10.1186/cc13895

**Published:** 2014-05-27

**Authors:** Kazuyoshi Aoyama, P Gareth Seaward, Stephen E Lapinsky

**Affiliations:** 1 Intensive Care Unit, Mount Sinai Hospital, 600 University Avenue, Toronto, ON M5G1X5, Canada; 2 Interdepartmental Division of Critical Care, Faculty of Medicine, University of Toronto, 1 Kings College Circle, Toronto, ON M5S1A8, Canada; 3 Department of Obstetrics and Gynecology, Mount Sinai Hospital, 600 University Avenue, Toronto, ON M5G1X5, Canada

## Abstract

Management of the critically ill pregnant woman is complicated by potential adverse effects of both maternal illness and ICU interventions on the fetus. This paper reviews the potential risks to the fetus of maternal critical illness, including shock, hypoxemia, and fever, as well as the effects of critical care management, such as drug therapy and radiological investigations. The authors’ recommended approach to management is provided. Prior publications and new data presented identify that there is insufficient information to prognosticate accurately on fetal outcome after maternal critical illness, although maternal shock, hypoxemia and early gestational age are likely significant risk factors.

## Introduction

Management of the critically ill pregnant patient is complicated by the presence of the fetus. The fetus is at risk from the effects of the maternal illness as well as from the effects of critical care interventions. Altering maternal care to protect the fetus (for example, avoiding certain drugs or radiological investigations) may have deleterious effects on both mother and fetus. Limited data exist to identify fetal risk factors in critically ill mothers admitted to the ICU. This article reviews the potential adverse effects of maternal critical illness on the fetus, as well as the limited data available to predict fetal outcome. We provide data from a secondary analysis of a database of critically ill pregnant women. The potential dilemma of termination of pregnancy to improve maternal well-being is also addressed.

## Potential effects of critical illness on the fetus

### Shock

Shock may occur in the pregnant patient as a result of hemorrhage, sepsis or embolism as well as other causes. Pregnancy results in dramatic changes to the uterine circulation, with a marked increase in blood flow, related to an overall increase in maternal cardiac output and a reduction in resistance of the vascular bed of the uteroplacental circulation [1]. Placental circulation is unique in having no capillary microcirculation but a high flow, low resistance system of spiral arteries. The uteroplacental vascular bed is usually maximally dilated, but has a strong alpha-adrenergic receptor system with a marked decrease in uteroplacental blood flow in response to endogenous or exogenous stimulation [1]. Normal maternal hemodynamic responses to hypotension are therefore not protective of the fetus, but rather protect maternal vital organs, shifting cardiac output from the uterine and placental circulation to the maternal brain and heart. Fetal oxygenation decreases as placental perfusion (and therefore oxygen delivery) decreases [2].

In the critically ill pregnant patient, several hemodynamic factors may potentially adversely affect the fetus. These include maternal shock (for example, sepsis, hemorrhage, pulmonary embolism) or hypotension due to sedative drugs or positive pressure ventilation. Although beneficial in reversing maternal hypotension, the use of sympathomimetic agents may reduce placental perfusion. Alpha-adrenergic agents directly vasoconstrict the uteroplacental vascular bed and beta-adrenergic agents dilate other vascular beds, shunting blood away from the uterus [1]. Clinical data on the use of these agents is limited, although animal studies provide some information. Norepinephrine, epinephrine and dopamine have all been shown to reduce uterine blood flow [3]. Ephedrine and phenylephrine have been used for maternal hypotension secondary to neuraxial anesthesia, in small bolus doses or by infusion. Phenylephrine is currently the preferred option [4,5]. Left lateral tilt of the hypotensive pregnant women should always be considered as an early intervention, to reverse a supine hypotensive effect [6]. Our practice with regard to vasopressor use in pregnancy is to follow our usual protocols (that is, most commonly norepinephrine with or without dobutamine) after adequate fluid resuscitation and left lateral positioning. In consultation with the obstetric team electronic fetal heart rate monitoring (see below) may be utilized to identify whether these infusions are producing beneficial or adverse effects on the fetus.

### Hypoxemia

Oxygen delivery to the fetus is dependent on utero-placental blood flow and blood oxygen content. Fetal oxygenation is precarious with partial pressure of oxygen in the umbilical vein (returning from the placenta to the fetus) of about 28 mmHg [7]. The oxygen delivery to fetal organs is increased by the left-shifted oxygen dissociation curve of fetal hemoglobin and high fetal cardiac output. As described in the section above, maternal shock will significantly reduce the oxygen delivery to the placenta and fetus. Maternal hypoxia causes hypoxic fetoplacental vasoconstriction, reducing placental flow and reducing fetal oxygen transfer [8]. A decrease in maternal oxygen saturation is likely to cause a significant drop in fetal oxygen delivery [7]. A theoretical model based on animal studies suggests that a decrease in maternal arterial oxygen saturation from 96% to 85% would be associated with a decrease in fetal umbilical vein oxygenation from about 70% to 55% [7]. If the oxygen content of fetal blood is reduced (even by up to 50%), compensation can occur as the fetus redirects cardiac output to the fetal heart and brain [9]. A further drop in oxygen content results in anerobic metabolism and if oxygen delivery is reduced by more than 75%, central nervous system damage may ensue.

Reliable human data are not available to confirm the potential adverse effects of acute maternal hypoxia on the fetus. A study in non-critically ill pregnant women generated transient maternal hypoxemia (mean saturation <85%) by inhalation of 10% oxygen. Redistribution of maternal blood flow was noted favoring the maternal brain, but no adverse effects were noted on fetal monitoring [10]. A report of six pregnant women admitted to the ICU with severe acute respiratory distress syndrome (ARDS) describes maternal oxygen saturations initially in the range of 50 to 88% [11]. Despite aggressive ICU care there were only two good neonatal outcomes, with three intrauterine or neonatal deaths and one neonate surviving with hypoxic-ischemic encephalopathy. However, although autopsy in one neonate confirmed multiorgan hypoxic injury, other insults to the fetus may have played a role. A correlation between the degree of maternal hypoxemia and fetal outcome was not demonstrated.

Our practice is to aim for a maternal oxygen saturation greater than 92% when possible, although evidence to support this threshold is lacking. Consideration needs to be given to the potential adverse effects of such a cutoff - for example, the higher positive end-expiratory pressure (PEEP) required may decrease cardiac output and adversely affect placental blood flow. In the presence of severe ARDS with intractable hypoxemia, consideration may be given to delivery in collaboration with the obstetrician, if this is considered beneficial to the fetus (see also the section below, ‘Terminating pregnancy for ‘maternal well-being’’).

### Fever

The management of fever is not always considered essential in the stable ICU patient, and may be harmful in patients with sepsis [12]. However, for pregnant patients, high body temperature may have adverse structural and functional effects on the fetus. A number of fetal effects have been described, which include neurological (including autism) as well as craniofacial and cardiac defects [13]. The limited data available do not permit identification of a clear temperature threshold for risk. Fetal risk increases linearly with duration of the elevated temperature and exponentially with the degree of temperature elevation, an elevation of 2°C above normal for 24 hours likely conferring risk [13]. Fever should be rapidly treated with antipyretic agents (for example, acetominophen) and external cooling.

### Cardiac arrest

The frequency of cardiac arrest in pregnancy varies from 1 in 20,000 to 1 in 50,000 pregnancies [14,15]. The limited cardiac output provided by cardiopulmonary resuscitation (CPR) may have devastating effects on the fetus.

The usual management of cardiac arrest requires some modification in the pregnant patient, particularly related to patient positioning and emergency cesarean delivery [16]. Aortocaval compression by the gravid uterus may adversely affect maternal hemodynamics; this occurs from approximately 20 weeks gestation [16]. Manual uterine displacement to the left while maintaining supine position is better than left-lateral tilt in terms of optimizing the quality of CPR [16,17]. Emergency cesarean section may have dramatic beneficial effects on maternal hemodynamics and may be life-saving for the fetus (if viable). If return of spontaneous circulation does not occur by 4 minutes of resuscitative efforts, or if the mother has sustained an obviously non-survivable injury, immediate emergency cesarean delivery should be considered [18]. Optimally, delivery should occur within 5 minutes of cardiac arrest. Relief of aortocaval compression after the uterus has been emptied can result in a 60 to 80% increase in maternal cardiac output [19]. One review evaluated emergent cesarean deliveries for pregnant women with cardiac arrest and identified several case reports, including 38 cases in total, in which 34 infants (25 to 42 weeks gestation) survived and 4 other infants survived initially, but died several days later from complications of prematurity and anoxia [18]. Notably, 9 of 12 infants delivered within 5 minutes had a normal neurological outcome, although neonatal outcome was not reported for the other three patients. Twenty mothers were considered to have potentially reversible conditions, of whom 13 were discharged home in good condition. In no cases was there a deterioration in maternal condition related to the cesarean section, and in many a dramatic improvement in hemodynamic status was noted.

## Potential effects of ICU management on the fetus

### Drug therapy

The potential risks of vasoactive drugs on placental perfusion have been described in the section on maternal shock above. Despite these potential effects, maintenance of maternal blood pressure with these agents may be essential for maternal and fetal survival.

Teratogenicity concerns should not influence drug therapy in the critically ill mother, although the clinical team should be cognizant of the concerns of family members in this regard. The risk of teratogenesis for the fetus in normal pregnancy is approximately 1 to 3% [20]. The risk is highest in the first trimester, and observational studies have investigated the risk of fetal teratogenicity in first trimester exposure of pregnant women to surgery and anesthesia [21,22]. No increased risk of fetal teratogenesis was observed, but the exposure to sedatives and analgesics was limited to a short period in the operating room.

Evidence to support decisions regarding sedation and analgesia in the ICU is limited, and no guidelines are available. An increased risk of cleft lip and plate with benzodiazepine use in early pregnancy has been demonstrated in an animal model [23], but has not been confirmed in humans [24]. Of the benzodiazepines, transfer to the fetus is likely lowest with midazolam [25]. Most sedatives, including propofol and thiopental, can decrease blood pressure and therefore utero-placental blood flow. A single report describes two cases of acidosis occurring in pregnant women undergoing a prolonged infusion of propofol for neurosurgical procedures [26]. Opioids, including morphine and fentanyl, have not been associated with fetal teratogenicity. Although most non-depolarizing agents can cross to the placenta, they are unlikely to have a clinical impact on the fetus. The concentration ratio between the umbilical vein and maternal vein for muscle relaxants ranges from 7 to 26% [27].

Our practice with regard to sedation and analgesia in the pregnant woman is similar to the non-pregnant patient, utilizing an approach with which we have significant experience in the non-pregnant patient. We utilize a protocol based on a sedation scale to minimize drug use [28], using fentanyl for analgesia and midazolam for sedation, when required. It is important to plan for neonatal respiratory depression in the event of delivery in the sedated mother.

### Radiological investigations

Radiologic investigations are often essential for the evaluation and management of the critically ill pregnant patient. While there are potential risks of exposing the fetus to ionizing radiation, maternal recovery is essential for fetal well-being, and appropriate radiologic procedures should not be avoided [29]. Fetal exposure may be reduced by shielding the abdomen with lead and using a well-collimated X-ray beam. Estimated fetal radiation exposure can be limited to safe levels for most procedures using these techniques, although investigations such as abdominal-pelvic computed tomography (CT) cannot avoid significant fetal radiation exposure (Table 1) [29,30].

**Table 1:** Risk of fetal radiation exposure resulting from radiologic studies in the critically ill pregnant patient

	**Fetal radiation exposure (mGy)**
**Investigation**	
**Chest X-ray (with abdomen shielded)**	0.01
**Ventilation-perfusion scan**	
**Perfusion**	0.1-1
**Ventilation**	0.1-0.4
**CT pulmonary angiogram**	0.1-4
**CT pelvis and abdomen**	30-50
**Radiation effect on the fetus**	
**Teratogenicity**	50-100
**Oncogenicity**	20-50

CT, computed tomography.

The adverse effects of exposure of the fetus to radiation include oncogenicity and teratogenicity. A doubling of the risk of childhood leukemia may result from fetal exposure in the range of 20 to 50 mGy (2 to 5 rads). Most ICU investigations can be carried out well below these levels of exposure to the fetus (Table 1). Teratogenicity appears to occur at higher radiation doses, in the range of 50 to 100 mGy (5 to 10 rads). Recently, Ray and colleagues [31] conducted a population-based study of 1.8 million maternal-child pairs to investigate the association between fetal exposure to CT and radionuclide imaging, and the risk of childhood cancer. The absolute annual risk of childhood malignancy is about 1 in 10,000, and the risk did not appear to be increased following *in utero* radiation exposure (hazard ratio 0.68; 95% confidence interval 0.25 to 1.80).

In the critically ill mother, radiological investigations should be performed as required, and not withheld if there is a likelihood of benefitting the mother. Every effort should nonetheless be made to minimize uterine exposure, particularly in the first trimester. Chests X-rays are performed as required (but not as a routine; for example, not daily) and chest CT when required for diagnosis of pulmonary embolism or for evaluation of pulmonary parenchyma. In the relatively rare situation of intrabdominal catastrophe, CT of the abdomen and pelvis may be necessary, despite the clear risk of fetal exposure.

## Fetal monitoring

Continuous electronic monitoring of fetal heart rate (FHR) can be used to identify changes in fetal physiology as reflected by normal or abnormal fetal heart rate patterns [31]. Abnormal patterns of FHR have been identified that have good sensitivity but varying specificity for fetal distress. Fetal tachycardia often occurs in the presence of maternal infection or chorioamnionitis, but more ominous is the finding of fetal bradycardia or sinusoidal variation in FHR, which may suggest fetal hypoxia. In the event of spontaneous labor, the FHR changes during uterine contractions may be informative; early decelerations imply bradycardia coinciding with contractions, and are benign. In contrast, repetitive late decelerations, with bradycardia beginning beyond the peak in contraction and persisting after the contraction, may be a sign of fetal compromise, particularly if associated with a reduced beat-to-beat variation. This abnormal fetal physiology is usually a result of uteroplacental or fetal pathology, but in the critically ill mother may result from maternal hypoxemia or reduced cardiac output producing reduced uterine oxygen delivery. FHR monitoring requires expertise to interpret, and appropriate obstetric support if delivery is warranted on the basis of suspected fetal compromise. Our practice is to perform intermittent monitoring (for example, every 12 hours) by an obstetric nurse. In some situations, continuous monitoring is performed, with the continuous presence of an obstetric nurse to allow interpretation and prompt and appropriate intervention.

Ultrasound-based biophysical profile evaluation provides another method of fetal assessment [32]. Ultrasound is used to evaluate factors such as spontaneous movement, breathing action, and amniotic fluid volume. A normal biophysical profile usually carries a good prognosis. The biophysical profile can be supplemented with fetal Doppler studies of the umbical artery as well as the fetal middle cerebral artery. Results from fetal Doppler help to guide decisions around intervention for fetal compromise.

## Fetal outcome after maternal critical illness

In a systematic review of 40 studies of pregnant and postpartum women admitted to the ICU, the proportion of patients who were pregnant on ICU admission was median 16% (interquartile range (IQR) 6 to 28%) [33]. Perinatal mortality was reported inconsistently, but data were available in 22 of the studies, with a median mortality of 20% (IQR 11 to 32%). Only one study to date has specifically evaluated fetal prognostic predictors in this patient group, and assessed only patients with non-obstetrical causes for ICU admission at a single center [34]. In 93 pregnant women, 32 fetal losses occurred with 10 neonates requiring ICU care. Multivariable logistic regression analysis identified three factors associated with fetal loss, namely maternal shock (odds ratio 6.85), maternal transfusion of blood products (odds ratio 7.24) and lower gestational age (odds ratio 1.2 per week less than 37).

We performed a secondary analysis of 30 pregnant patients requiring intensive care, extracted from a database of 332 pregnant and postpartum patients who were at more than 12 weeks gestation, and admitted to the ICU for more than 24 hours [35]. The definition of fetal death in this database included only deaths occurring from the time of the mother’s ICU admission to hospital discharge. Thirty pregnant patients (9% of the 332 patients in the database) were identified, comprising 24 patients in whom the fetus survived and 6 patients in whom the fetus died.

The characteristics of the mothers of the fetuses who survived were compared with those of fetuses that died, with respect to clinical data. Sixty-four variables were compared between the two groups and no adjustment was made for the number of tests done. Data are summarized by median and IQR for continuous data and by frequencies and proportions for categorical variables. Fisher’s exact test and Wilcoxon rank-sum test were used and *P* < 0.05 was considered statistically significant. SAS system version 9.1 for Windows (SAS Institute, Inc., Cary, NC, USA) was used to analyze the data.

Almost all patients (90%) were admitted to ICU with medical conditions. Seventeen of these patients (56%) delivered during their ICU stay. Two women died in the ICU (7%), one from hypoxic respiratory failure due to pneumonia and Churg-Strauss syndrome, and the other due to septic shock. Both maternal deaths were preceded by fetal death. Underlying maternal conditions and maternal and fetal outcome are shown in Table 2. Half of the 30 patients required ventilatory support and two of four patients with shock required inotrope support. Nine patients (30%) had preterm delivery. Partial thromboplastin time was significantly lower, and length of stay in ICU was significantly longer in the group with fetal mortality (Table 3). Several parameters, including lower gestational age, maximum temperature, low alkaline phosphatase, low serum bicarbonate and high Acute Physiology and Chronic Health Evaluation II showed a trend towards association with fetal mortality, but not reaching statistical significance. No difference was found for some parameters considered important *a priori*, namely oxygenation, blood pressure and hemoglobin level.

**Table 2:** Characteristics and outcome of mothers and neonates

**ICU admission problem**	**Specific conditions**	**Number**	**Preterm delivery (n)**	**Fetal mortality (n)**	**Maternal mortality (n)**
**Respiratory**	Churg-Strauss, asthma, pneumonia, pneumothorax, ARDS	12	4	3	1
**Neurological**	SAH, intracerebral hemorrhage, TBI, seizure	7	3	3	1
**Infectious**	Pyelonephritis, pneumonia	6	0	0	0
**Cardiac**	Hypertensive crisis	3	1	0	0
**Hematological**	Lymphoma	1	1	0	0
**Liver**	Fulminant hepatic failure	1	0	0	0

ARDS, acute respiratory distress syndrome; SAH, subarachnoid hemorrhage; TBI, traumatic brain injury.

**Table 3:** Comparison of maternal clinical parameters and laboratory results during first 24 hours of ICU admission

**Variables**	**Fetus survived, median (IQR) (N = 24)**	**Fetus died, median (IQR) (N = 6)**	***P*-value**
**Age (years)**	25.5 (19.5-33.0)	24.0 (19.0-28.0)	0.46
**Gestational age (weeks)**	30.5 (25.5-35.5)	20.5 (18.0-29.0)	0.05
**Gravity**	1 (0–1.5)	0 (0–1)	0.31
**APACHE II**	12.5 (7.5-14.0)	15.5 (13.0-21.0)	0.08
**GCS**	15 (14–15)	12 (7–15)	0.13
**Maximum temperature (celsius)**	37.5 (36.8-38.1)	38.6 (37.6-39.5)	0.07
**Minimum systolic blood pressure (mmHg)**	107 (103–118)	108 (88–110)	0.80
**Minimum diastolic blood pressure (mmHg)**	58 (55–68)	69 (61–70)	0.41
**P/F ratio**	238 (178–375)	190 (177–292)	0.44
**A-a DO2**	164 (62–236)	172 (127–374)	0.27
**Alkaline phosph**a**tase (U/L)**	160 (99–251)	57.5 (44.5-91.5)	0.05
**Hgb (g/dL)**	96.5 (85–105.5)	91 (74–97)	0.28
**Bicarbonate (mmol/L)**	22 (18.0-23.6)	18.5 (15.5-20.1)	0.06
**PTT (seconds)**	28.7 (25.9-32.4)	20.6 (16.6-24.5)	0.03
**Hospital LOS (days)**	7.5 (7–11)	11 (9–31)	0.25
**ICU LOS (days)**	2 (2–4)	7.5 (5–9)	<0.002

A-a DO2, alveolar-arterial oxygen difference; APACHE, Acute Physiology and Chronic Health Evaluation; GCS, Glasgow coma scale; Hgb, hemoglobin; IQR, interquartile range; LOS, length of stay; P/F, partial pressure of oxygen to fraction of inspired oxygen ratio; PTT, partial thromboplastin time.

The previously published data and our analysis demonstrate that there is insufficient evidence to confidently prognosticate with regard to fetal outcome in the critically ill pregnant woman. Maternal shock and lower gestational age are clear risk factors, and although not identified in these studies, severe maternal hypoxemia likely carries an additional risk.

## Terminating pregnancy for ‘maternal well-being’

Due to the effects of respiratory physiological changes of late pregnancy and the fetal demands for oxygen consumption and carbon dioxide excretion, delivery of the pregnant patient with respiratory failure may result in improvement in the mother’s condition [36]. However, data from small case series addressing this issue have not consistently demonstrated a significant benefit to the mother, and delivery carries a risk of harm [37,38]. Although some degree of improvement in oxygenation has been noted [36], this is not associated with improvement in respiratory system compliance or PEEP level [37]. Delivery should therefore not be performed purely in the hope of improving maternal condition, at the expense of the fetus. However, if the fetus is at a viable gestation but is at risk due to severe maternal hypoxia, consultation with the perinatal team may identify a benefit to the fetus of early delivery. Obstetric indications should determine the mode of delivery. While cesarean section allows more rapid delivery in the critically ill patient, the increased physiological stress of operative delivery may carry increased risks for the mother.

## Conclusion

The fetus of a critically ill pregnant women is at risk of adverse effects related to both the maternal illness as well as the ICU treatment and interventions. Data on these risks are limited, but from the underlying physiology, animal studies and limited human case series, several factors likely contribute, namely maternal shock, hypoxemia and fever, drug therapy, radiological investigations, lower gestational age and maternal transfusion. It is nevertheless important to stress that interventions and drugs essential to treat the critically ill mother should not be withheld due to concern for their potential adverse effects on the fetus.

## Abbreviations

ARDS: Acute respiratory distress syndrome; CPR: Cardiopulmonary resuscitation; CT: Computed tomography; FHR: Fetal heart rate; IQR: Interquartile range; PEEP: Positive end-expiratory pressure.

## Competing interests

The authors declare that they have no competing interests.

## Authors’ contributions

KA and SEL conceived this paper and performed the literature review. KA performed the analysis on the pre-existing database, and SEL and PGS provided input on the interpretation. KA wrote the initial draft of the manuscript, and PGS and SEL helped draft the final version, which was approved by all authors.

## References

[B1] Assali NS Dynamics of the uteroplacental circulation in health and disease Am J Perinatol 1989 6 105 109 10.1055/s-2007-999558 2540758

[B2] Wilkening RB Meschia G Effect of occluding one umbilical artery on placental oxygen transport Am J Physiol 1991 260 H1319 H1325 1901461 10.1152/ajpheart.1991.260.4.H1319

[B3] James FM 3rd Greiss FC Jr Kemp RA An evaluation of vasopressor therapy for maternal hypotension during spinal anesthesia Anesthesiology 1970 33 25 34 10.1097/00000542-197007000-00010 5464319

[B4] Mercier FJ Augè M Hoffmann C Fischer C Le Gouez A Maternal hypotension during spinal anesthesia for caesarean delivery Minerva Anestesiol 2013 79 62 73 23135692

[B5] Habib AS A review of the impact of phenylephrine administration on maternal hemodynamics and maternal and neonatal outcomes in women undergoing cesarean delivery under spinal anesthesia Anesth Analg 2012 114 377 390 10.1213/ANE.0b013e3182373a3e 22104076

[B6] Kinsella SM Lohmann G Supine hypotensive syndrome Obstet Gynecol 1994 83 774 788 8164943

[B7] Meschia G Fetal oxygenation and maternal ventilation Clin Chest Med 2011 32 15 19 10.1016/j.ccm.2010.11.007 21277445

[B8] Hampl V Jakoubek V Regulation of fetoplacental vascular bed by hypoxia Physiol Res 2009 58 Suppl 2 S87 S93 20131940 10.33549/physiolres.931922

[B9] Peeters LL Sheldon RE Jones MD Jr Makowski EL Meschia G Blood flow to fetal organs as a function of arterial oxygen content Am J Obstet Gynecol 1979 135 637 646 507116 10.1016/s0002-9378(16)32989-1

[B10] Polvi HJ Pirhonen JP Erkkola RU The hemodynamic effects of maternal hypo- and hyperoxygenation in healthy term pregnancies Obstet Gynecol 1995 86 795 799 10.1016/0029-7844(95)00260-X 7566851

[B11] Oluyomi-Obi T1 Avery L Schneider C Kumar A Lapinsky S Menticoglou S Zarychanski R Perinatal and maternal outcomes in critically ill obstetrics patients with pandemic H1N1 influenza A J Obstet Gynaecol Can 2010 32 443 447 448–452 20500952 10.1016/S1701-2163(16)34497-8

[B12] Lee BH Inui D Suh GY Kim JY Kwon JY Park J Tada K Tanaka K Ietsugu K Uehara K Dote K Tajimi K Morita K Matsuo K Hoshino K Hosokawa K Lee KH Lee KM Takatori M Nishimura M Sanui M Ito M Egi M Honda N Okayama N Shime N Tsuruta R Nogami S Yoon SH Fujitani S Association of body temperature and antipyretic treatments with mortality of critically ill patients with and without sepsis: multi-centered prospective observational study Crit Care 2012 16 R33 22373120 10.1186/cc11211 PMC3396278

[B13] Edwards MJ Review: Hyperthermia and fever during pregnancy Birth Defects Res A Clin Mol Teratol 2006 76 507 516 10.1002/bdra.20277 16933304

[B14] Benhamou D Chassard D Mercier FJ Bouvier-Colle MH The seventh report of the confidential enquiries into maternal deaths in the United Kingdom: comparison with French data Ann Fr Anesth Reanim 2009 28 38 43 10.1016/j.annfar.2008.11.002 19101110

[B15] Dijkman A Huisman CM Smit M Schutte JM Zwart JJ van Roosmalen JJ Oepkes D Cardiac arrest in pregnancy: increasing use of perimortem caesarean section due to emergency skills training? BJOG 2010 117 282 287 10.1111/j.1471-0528.2009.02461.x 20078586

[B16] Vanden Hoek TL Morrison LJ Shuster M Donnino M Sinz E Lavonas EJ Jeejeebhoy FM Gabrielli A Part 12: Cardiac arrest in special situations: 2010 American Heart Association guidelines for cardiopulmonary resuscitation and emergency cardiovascular care Circulation 2010 122 S829 S861 10.1161/CIRCULATIONAHA.110.971069 20956228

[B17] Kundra P Khanna S Habeebullah S Ravishankar M Manual displacement of the uterus during caesarean section Anaesthesia 2007 62 460 465 10.1111/j.1365-2044.2007.05025.x 17448057

[B18] Katz V Balderston K DeFreest M Perimortem cesarean delivery: were our assumptions correct? Am J Obstet Gynecol 2005 192 1916 1920 discussion 1920–1921 10.1016/j.ajog.2005.02.038 15970850

[B19] Hill CC Pickinpaugh J Trauma and surgical emergencies in the obstetric patient Surg Clin North Am 2008 88 421 440 viii 10.1016/j.suc.2007.12.006 18381121

[B20] Honein MA Paulozzi LJ Cragan JD Correa A Evaluation of selected characteristics of pregnancy drug registries Teratology 1999 60 356 364 10.1002/(SICI)1096-9926(199912)60:6<356::AID-TERA8>3.0.CO;2-B 10590397

[B21] Duncan PG Pope WD Cohen MM Greer N Fetal risk of anesthesia and surgery during pregnancy Anesthesiology 1986 64 790 794 10.1097/00000542-198606000-00019 3717642

[B22] Mazze RI Kallen B Reproductive outcome after anesthesia and operation during pregnancy: a registry study of 5405 cases Am J Obstet Gynecol 1989 161 1178 1185 10.1016/0002-9378(89)90659-5 2589435

[B23] Safra MJ Oakley GP Jr Association between cleft lip with or without cleft palate and prenatal exposure to diazepam Lancet 1975 2 478 480 51287 10.1016/s0140-6736(75)90548-6

[B24] Rosenberg L Mitchell AA Parsells JL Pashayan H Louik C Shapiro S Lack of relation of oral clefts to diazepam use during pregnancy N Engl J Med 1983 309 1282 1285 10.1056/NEJM198311243092103 6633586

[B25] Briggs GG Freeman RK Yaffe SJ Drugs in Pregnancy and Lactation. A Reference Guide to Fetal and Neonatal Risk 2008 8 Philadelphia, PA: Lippincott Williams & Wilkins

[B26] Hilton G Andrzejowski JC Prolonged propofol infusions in pregnant neurosurgical patients J Neurosurg Anesthesiol 2007 19 67 68 10.1097/ANA.0b013e31802b31c8 17198107

[B27] Guay J Grenier Y Varin F Clinical pharmacokinetics of neuromuscular relaxants in pregnancy Clin Pharmacokinet 1998 34 483 10.2165/00003088-199834060-00004 9646009

[B28] Mehta S Burry L Cook D Fergusson D Steinberg M Granton J Herridge M Ferguson N Devlin J Tanios M Dodek P Fowler R Burns K Jacka M Olafson K Skrobik Y Hébert P Sabri E Meade M SLEAP Investigators; Canadian Critical Care Trials Group Daily sedation interruption in mechanically ventilated critically ill patients cared for with a sedation protocol: a randomized controlled trial JAMA 2012 308 1985 1992 10.1001/jama.2012.13872 23180503

[B29] Ratnapalan S Bentur Y Koren G Doctor, will that x-ray harm my unborn child? CMAJ 2008 179 1293 1296 10.1503/cmaj.080247 19047611 PMC2585137

[B30] Lowe SA Diagnostic radiography in pregnancy: risks and reality Aust N Z J Obstet Gynaecol 2004 44 191 196 10.1111/j.1479-828X.2004.00212.x 15191441

[B31] Ray JG Schull MJ Urquia ML You JJ Guttmann A Vermeulen MJ Major radiodiagnostic imaging in pregnancy and the risk of childhood malignancy: a population-based cohort study in Ontario PLoS Med 2010 7 e1000337 10.1371/journal.pmed.1000337 20838660 PMC2935460

[B32] Moaveni DM Birnbach DJ Ranasinghe JS Yasin SY Fetal assessment for anesthesiologists: are you evaluating the other patient? Anesth Analg 2013 116 1278 1292 10.1213/ANE.0b013e31828d33c5 23558831

[B33] Pollock W Rose L Dennis CL Pregnant and postpartum admissions to the intensive care unit: a systematic review Intensive Care Med 2010 36 1465 1474 10.1007/s00134-010-1951-0 20631987

[B34] Cartin-Ceba R Gajic O Iyer VN Vlahakis NE Fetal outcomes of critically ill pregnant women admitted to the intensive care unit for nonobstetric causes Crit Care Med 2008 36 2746 2751 10.1097/CCM.0b013e318186b615 18828192

[B35] Lapinsky SE Hallett D Collop N Drover J Lavercombe P Leeman M Moola S Paruk F Bernstein M Moodley J Evaluation of standard and modified severity of illness scores in the obstetric patient J Crit Care 2011 26 535.e1 535.e7 21106337 10.1016/j.jcrc.2010.10.003

[B36] Daily WH Katz AR Tonnesen A Allen SJ Beneficial effect of delivery in a patient with adult respiratory distress syndrome Anesthesiology 1990 72 383 386 10.1097/00000542-199002000-00027 2301770

[B37] Tomlinson MW Caruthers TJ Whitty JE Gonik B Does delivery improve maternal condition in the respiratory-compromised gravida? Obstet Gynecol 1998 91 108 111 10.1016/S0029-7844(97)00585-1 9464731

[B38] Mabie WC Barton JR Sibai BM Adult respiratory distress syndrome in pregnancy Am J Obstet Gynecol 1992 167 950 957 10.1016/S0002-9378(12)80018-4 1415431

